# Social class and moral judgment: a process dissociation perspective

**DOI:** 10.3389/fsoc.2024.1391214

**Published:** 2024-04-30

**Authors:** Andreas Tutic, Friederike Haiser, Ivar Krumpal

**Affiliations:** ^1^Department of Sociology, University of Bergen, Bergen, Norway; ^2^Institute of Sociology, Leipzig University, Leipzig, Lower Saxony, Germany

**Keywords:** social class, empathy, cognitive style, utilitarian judgment, deontological judgment, process dissociation

## Abstract

Do social classes differ in moral judgment? Previous research showed that upper-class actors have a greater inclination toward utilitarian judgments than lower-class actors and that this relationship is mediated by empathic concern. In this paper, we take a closer look at class-based differences in moral judgment and use the psychometric technique of process dissociation to measure utilitarian and deontological decision inclinations as independent and orthogonal concepts. We find that upper-class actors do indeed have a greater inclination toward decisions consistent with utilitarian principles, albeit only to a quite small extent. Class-related differences are more pronounced with respect to deontological judgments, in so far as upper-class actors are less inclined to judgments consistent with deontological principles than lower-class actors. In addition, it is shown that class-based differences in utilitarian judgments are mediated by cognitive styles and not so much by empathic concern or moral identity. None of these potential mediators explains class-based differences in the inclination toward deontological judgments.

## 1 Introduction

In practical philosophy as well as in moral psychology, scholars differentiate between utilitarianism (Mill, 1863) and deontology (Kant, 1797). While utilitarianism is outcome-oriented focusing on the consequences of behaviors in terms of wellbeing, happiness and the maximization of benefit for the individual and the society, deontological approaches evaluate behaviors according to their intrinsic qualities in terms of moral rights and duties (Greene et al., 2001; Conway and Gawronski, 2013). From the perspective of deontological ethics, the morality of actions is determined by their intrinsic nature, regardless of outcome, and there is a categorical imperative to avoid proscriptions like killing as universalizing the proposition renders it illogical, and it violates fundamental dignity (Kant, 1797).

Studying the extent and the conditions under which actors lean toward utilitarian or deontological judgment is vital, since moral reasoning and moral emotions are among the fundamental driving forces of human behavior (Greene et al., 2001, 2004, 2008; Haidt, 2001, 2003). For instance, Tutić et al. (2022) show that (hypothetical) triage decisions in the context of the COVID-19 pandemic are affected by respondents' utilitarian and deontological decision inclinations. On the societal level, divergencies between utilitarian and deontological moral judgment are often at the very core of long-standing controversies in public discourse (Greene, 2013). Consider for example the question of whether abortion is morally acceptable. For many deontologists, abortion is never morally acceptable, because it violates the unborn's fundamental right to life (Thomson, 1971; Tooley, 1972). From an utilitarian point of view, the question of whether an abortion is morally legitimate depends on the details of the case under consideration (Singer, 1980). If the abortion implies a net loss of potential happiness experienced by the society as whole, utilitarians will oppose it. However, there are also cases in which society gains from an abortion and hence utilitarians endorse it, for example in some cases in which the birth of the infant implies the death of the mother (Greene, 2013).

In behavioral science, moral judgment is typically studied via confronting subjects with hypothetical scenarios, so-called moral dilemmas, which to a smaller or greater extent bring deontological principles and utilitarian principles in conflict with another (e.g., Petrinovich et al., 1993; Greene et al., 2001, 2004, 2008; Nichols, 2002; Mendez et al., 2005).

Classic examples of moral dilemmas are the footbridge dilemma (Thomson, 1986) and the trolley dilemma (Foot, 1967). In the footbridge dilemma, a runaway trolley is about to run over five workers and kill them. They can be saved by pushing another person off a pedestrian bridge and into the path of the trolley. In the trolley dilemma, the lives of the five workers can be saved by hitting a switch that will turn the trolley onto an alternate set of tracks where it will kill one person. Since the consequences in terms of saved lives are identical in both scenarios, utilitarians have no clear-cut reason to differentiate between these two dilemmas and typically find both the pushing of the pedestrian as well as the hitting of the switch as morally appropriate. In contrast, deontologists spot a subtle yet, in their reasoning, crucial difference between the two scenarios: In the footbridge dilemma, the person killed is not valued, as Kant (1797) demands, as an end for himself or herself, but treated as a means to achieve an independent end, whereas the death of the single worker in the trolley dilemma constitutes a tragic side effect (Greene et al., 2001, p. 2,205). As a consequence, deontologists often agree with hitting the switch in the trolley dilemma, but oppose pushing the person in the footbridge dilemma (Greene et al., 2001, 2004, 2008).

Beyond philosophical principles and moral theory, empirically observed decision tendencies can be explained by means of psychological processes (Kahane et al., 2015; Conway et al., 2018). For example, the original dual process model argues that “harm-rejection decision tendencies,” which align with deontological theories, reflect emotional reactions to the thought of causing harm, and “outcome-maximization decision tendencies,” which accord with utilitarian principles, reflect cognitive processing about outcomes. In comparison to the trolley dilemma, the footbridge dilemma is supposed to feel more emotionally evocative which motivates increased rejection of sacrificial harm, which can be interpreted as decisions consistent with deontological principles.

Côté et al. (2013) confront respondents with both the footbridge as well as the trolley dilemma as well as an allocation task to study the question of whether social classes differ in moral judgment. They find that upper-class individuals have a greater probability than lower-class individuals of evaluating the pushing of the person in the footbridge dilemma as morally appropriate. Côté et al. (2013) also find social classes do not differ regarding moral judgment in the trolley dilemma and interpret these findings to indicate that upper-class actors have a greater tendency toward utilitarian judgments than lower-class actors. In addition, they argue on theoretical grounds and show empirically that this relationship between social class and utilitarian judgments is partially mediated by differences in empathy.

In this paper, we follow the lead by Côté et al. (2013) and study the question of whether there are differences in moral judgment between social classes. Similarly to their pioneering work, we make use of moral dilemmas to measure subjects' utilitarian and deontological decision inclinations. However, we employ process dissociation (Jacoby, 1991; Payne and Bishara, 2009), a psychometric technique, which allows to measure subjects' inclinations toward utilitarian and deontological decisions independently from another (see Conway and Gawronski, 2013; Conway et al., 2018). This contrasts with the approach by Côté et al. (2013), which effectively assumes that utilitarian and deontological decision tendencies constitute opposing poles on a single continuum rather than orthogonal concepts.

Recall, Côté et al. (2013) find that social classes differ regarding moral judgment in the footbridge but not in the trolley dilemma. These differences, which emerge primarily on the more emotionally evocative footbridge dilemma, suggest manifestations of increased deontological decision tendencies primarily in more “personal” situations among people high in empathic concern, aversion to harming others, and other markers of affective reactions to harm (e.g., Conway and Gawronski, 2013; Reynolds and Conway, 2018). Since these dilemmas do differ substantially for people high in empathic concern and therefore stronger inclinations toward deontological judgments, but not for people low in empathic concern and therefore stronger inclinations toward utilitarian judgments, this finding suggests that social classes differ in their inclination toward deontological judgments but not necessarily in their inclination toward utilitarian judgments.

The fact that Côté et al. (2013) interpret their findings as evidence for more utilitarianism among higher-class individuals shows that they conceive a smaller inclination toward deontological judgments as logically identical to a greater inclination toward utilitarian judgments. It is precisely this assumption of a necessarily negative correlation between the inclinations toward utilitarian decision tendencies and deontological response inclinations the usage of process dissociation allows to abandon.

In addition, we also test empirically whether class-based differences in moral judgment are mediated by differences in emphatic concern. To expand on the existing literature, we consider additional potential mediators such as the tendency toward cognitive reflection (Frederick, 2005; Kahneman, 2011), the faith in the adequacy of first intuitions (Epstein et al., 1996) as well as the importance subjects place on having a moral identity (Aquino and Reed, 2002).

The remainder of the paper is structured as follows: Section 2 briefly reviews theoretical arguments as well as empirical findings from the literature, explains process dissociation, and formulates hypotheses. Section 3 provides information on our sample, methods, and variables. In Section 4 we describe our empirical findings. Section 5 concludes with a summary, discusses limitations of our approach, and provides directions for future research.

## 2 Theory, process dissociation, and hypotheses

### 2.1 Theoretical considerations

What explains class-based differences in moral judgment? Following the lead by Côté et al. (2013), we take a closer look at different mediating mechanisms that could potentially explain this relationship. To state it more technically: We identify mediating variables B explaining the relationship between independent variable A (social class) and dependent variable C (moral judgment). Following the logic of mediation analysis (Baron and Kenny, 1986; Hayes, 2022), we do so by arguing in theoretical as well as empirical terms why we expect both a correlation between A and B on the one hand and B and C on the other hand. We specify arguments as to why these relationships can be expected focusing on the following potential mediators: empathic concern, cognitive styles, and moral identity.^1^

*Empathic concern:* Why should social classes differ in moral judgment? Côté et al. (2013) argue that classes differ in moral judgment because they differ in empathic concern, which is negatively correlated with utilitarian judgment. Regarding the relationship between classes and empathy, Côté et al. (2013) rely on social-psychological literature regarding contextualism (cf. Kluegel and Smith, 1986; Kraus and Keltner, 2009; Kraus et al., 2010) and empathy (cf. Batson, 2011, 2014). Accordingly, due to living in an environment in which more existential risks are present and also due to a lack of material resources to deal with these risks, lower-class actors are more dependent on others than upper-class actors. Because empathy is instrumental in creating and maintaining social ties, lower-class actors in comparison to upper-class actors develop a greater tendency toward empathic concern as some kind of adaptive strategy in coping with unfavorable life circumstances. In line with this, Lammers et al. (2015) describe that increasing power leads people to focus more on themselves than on others, which is associated with lower empathy and compassion for others.

With respect to the hypothesized negative relationship between empathic concern and utilitarian decision tendencies, Côté et al. (2013) draw on Greene's dual-process model of moral judgment (Greene et al., 2001; Greene, 2007, 2013), which argues that deontological judgments are typically driven by “hot” and emotional moral intuitions, whereas utilitarian judgments mainly stem from “cold” and calculating moral reflections. Experimental research on moral dilemmas (Greene et al., 2001) as well as brain injured subjects (Mendez et al., 2005; Ciaramelli et al., 2007; Koenigs et al., 2007) quite consistently support the idea that deontological judgments are driven by emotional reactions. Along these lines, studies have shown that emotion regulation strategies (Lee and Gino, 2015; Li et al., 2017) and emotion regulation difficulties (Zhang et al., 2017) selectively influence deontological inclinations, while utilitarian inclinations remain unaffected. Against this background, Côté et al. (2013) reason that actors with greater empathic concern for others should be more susceptible to emotional responses to moral dilemmas and hence are supposed to be more likely to engage in deontological judgments. The idea that empathic concern influences moral judgment is also backed empirically by Maranges et al. (2021) who find that the association between lower deontological and utilitarian tendencies and unpredictability in childhood was statistically mediated by low levels of empathic concern and poor quality of social relationships. Summing up theoretical arguments as well as former empirical evidence, it can be hypothesized that empathic concern is a key mediator of the relationship between social class and moral inclinations.

*Cognitive styles:* In their exposition, Côté et al. (2013) focus on empathic concern as a mediator of the relationship between social class and moral inclinations. Taken their argument one step further, we argue that the dual-process model of moral judgment suggests that cognitive styles might function as another important mediator. Remember that a rather deliberative thinking style goes along with more utilitarian judgments, whereas a rather intuitive thinking style leads to more deontological judgments (Greene et al., 2001). Note that the argument underlying the proposed relationship between thinking styles and moral judgment is not that deontological judgment cannot be justified via reasoning; the impressive philosophical works of scholars such as Kant (1797) prove the opposite. What Greene's dual-process model posits is that the cost-benefit-analysis required for utilitarian judgment is a hallmark of reflective Type-2 processes, whereas deontological judgment is often driven by emotions. Further we argue that cognitive thinking styles are socially patterned. Various social theorists explain the development of a more rational or intuitive cognitive style as shaped by the exposition to different types of social and cultural influences (Dewey, 1933; Simmel, 1964; Bourdieu, 2000; Rivers et al., 2017). Brett and Miles (2021) provide evidence that upper-class actors exhibit more deliberative processing in comparison to lower-class actors. In particular, education consistently predicts preferences for deliberate processing. Higher education and social positions require independent and complex thinking, while economic disadvantages predict a greater reliance on intuitive thinking (Brett and Miles, 2021). Consistent therewith, studies show that economically disadvantaged people must focus their cognitive resources on obtaining basic necessities, which reduces the cognitive bandwidth available for other tasks and results in a rather automatic thinking style (Mani et al., 2013; Mullainathan and Shafir, 2013). Therefore, we argue that upper-class individuals tend to prefer a deliberative thinking style combined with utilitarian judgments, whereas lower-class individuals predominantly rely on automatic thinking along with deontological judgments. Focusing on our main research question which mediating mechanism is key in explaining the relationship between social class and moral inclinations, it can be argued that thinking dispositions and cognitive styles are plausible alternative hypotheses that could challenge the status of empathic concern as the most important mediator.

*Moral identity:* Expanding on the paper by Côté et al. (2013), we consider moral identity as another potential mediator of the relationship between social class and moral inclinations. Moral identity as a concept refers to an actor's internalized identity as a moral person as one possible component of a person's social self-schema (Aquino and Reed, 2002). There is a debate among researchers whether utilitarian judgments are the result of genuinely moral concerns, like a desire to maximize welfare, or an expression of a reduced concern over causing harm (see Baron and Spranca, 1997; Bazerman and Greene, 2010; Bennis et al., 2010). In contrast to findings arguing that only deontological, but not utilitarian, inclinations should be positively linked to an internalized moral identity (Bartels and Pizarro, 2011; Xu and Ma, 2016), research by Conway and Gawronski (2013) indicates that both deontological and utilitarian inclinations are positively correlated with a measure of moral identity internalization. Accordingly, Conway and Gawronski (2013) claim that both moral inclinations are considered genuine, inherent moral concerns. Against this background, it seems reasonable to expect a significant association between the weight that actors place on having a moral identity and utilitarian as well as deontological judgment.

Additionally, former research suggests that urban poverty goes along with fewer opportunities for developing a moral identity, e.g., commitment to moral projects (Hart et al., 1998; Hart and Matsuba, 2009), and even inhibits the evolvement of moral attitudes and values such as tolerance for divergent perspectives (Hart et al., 2006). Drawing on these findings, we argue that moral identity internalization should be related with social class as well as moral inclinations, thus constituting another potential mediator. To sum up, we hypothesize that differences in moral identity mediate the relationship between social class and moral inclinations. This constitutes another rival hypothesis challenging explanations and arguments in regards to mediation which are based on empathic concern or cognitive styles.

### 2.2 Process dissociation

As indicated, according to the theoretical argument by Côté et al. (2013) upper-class actors show a greater tendency toward utilitarian judgment than lower-class actors because they are less empathic and hence have a smaller tendency toward deontological judgment. Implicit in this argument is the idea that utilitarian and deontological decision tendencies constitute two opposing poles on a single continuum such that more utilitarian judgment necessarily goes hand in hand with less deontological judgment and vice versa.

This stance is also reflected in their methodological approach, which relies on comparing individual judgments in two moral dilemmas. In each dilemma, the subjects are described as performing protagonists who are asked to decide whether executing a certain harmful action in the given situation would be appropriate or inappropriate. On the one hand they make use of the footbridge dilemma (Thomson, 1986), a dilemma with high emotional arousal, and on the other hand they use the trolley dilemma (Foot, 1967), which involves few emotional affects (Greene et al., 2001). In the footbridge dilemma, respondents are described as located on the footbridge over the tracks and they can save the lives of workers by pushing a stranger off the bridge to stop a trolley heading toward the workers. In this case the stranger will die, and five workers will be saved. In contrast to that, in the trolley dilemma, respondents are described as at the wheel of a runaway trolley approaching a fork in the tracks. The trolley is driving toward five workers which the respondent can save by hitting a switch on the dashboard that will cause the trolley to change the direction accepting the death of a single worker. Since deontological judgments are often driven by emotional responses, people who make deontological decisions typically evaluate the action under consideration in the footbridge dilemma as inappropriate, whereas people whose decision inclinations align with utilitarian principles evaluate the action as appropriate. In contrast, both groups of people typically evaluate the action as appropriate in the trolley dilemma (Greene et al., 2001, 2008). Côté et al. (2013) find that social class has a greater effect on the probability of evaluating the action as appropriate in the footbridge than in the trolley dilemma. They interpret this finding to indicate that upper-class actors have a greater tendency toward utilitarian judgments than lower-class actors. However, a more natural interpretation of this finding is that upper-class actors are less inclined to deontological judgments than lower-class actors because the footbridge and the trolley dilemma differ in their appeal to people who prefer deontological decisions and not to people who prefer utilitarian decisions. Only if we adopt the assumption that utilitarian and deontological decision tendencies constitute opposing poles the preferred interpretation by Côté et al. (2013) attains plausibility.

However, recent research on moral inclinations using the psychometric technique of process dissociation (Jacoby, 1991; Kelley and Jacoby, 2000; Yonelinas, 2002; Payne and Bishara, 2009) has demonstrated that utilitarian and deontological response inclinations are not necessarily negatively correlated (Conway and Gawronski, 2013; Conway et al., 2018; Fleischmann et al., 2019).

The core idea of process dissociation in the domain of moral judgment is to confront a subject with a suitably designed set of moral dilemmas and to infer the subject's inclinations toward utilitarian vs. deontological decision making from the observed judgments. Key to the approach is the differentiation between congruent and incongruent dilemmas. If both groups, people who prefer deontological decisions as well as people who prefer utilitarian decisions, can be expected to evaluate the behavior under consideration as morally unacceptable, a moral dilemma is considered to be congruent. If people who prefer utilitarian decisions tend to judge the behavior under consideration as morally acceptable, while people who prefer deontological decisions judge the behavior as unacceptable, a moral dilemma is categorized as incongruent. For instance, consider the following example of a congruent moral dilemma:

“You are a surgeon. A young woman you know becomes pregnant, but she is not yet ready for children. She has not finished high school, has no income, and was abandoned by the father. If she has the baby now, she will be stuck as a single mother on welfare for the rest of her life. This will make things very hard on her and the baby. She thinks that it would be smarter to wait and have children later. So, although it is very difficult for her, she asks you to abort the baby. Is it appropriate for you to perform an abortion in order to let the mother live a better life?”

It is reasonable to argue that both people who make utilitarian decisions as well as people who make deontological decisions evaluate the abortion in this dilemma as morally inappropriate. For many subjects who are inclined to the deontological concern of rejecting harm, abortion is never morally appropriate because it violates the unborn's right to life (Greene, 2013, p. 309ff.). A respondent inclined to the utilitarian concern of maximizing aggregate happiness might also consider the abortion as morally inappropriate, because the loss of happiness to be experienced by the unborn is not necessarily outweighed by the additional hardship imposed on the mother. While both groups tend to agree in their judgment of congruent dilemmas, they often will disagree regarding incongruent dilemmas. For instance, consider the following incongruent dilemma:

“You are a surgeon. A young woman you know becomes pregnant, but her body reacts in an unusual fashion. She develops a severe case of preeclampsia, a dangerous syndrome that leads to rapid increases in blood pressure. The only treatment is to deliver the baby. Unless the baby is delivered soon, the mother will die. However, the baby is too young to survive on its own. If it is delivered, it will die. So, although it is very difficult for her, the mother asks you to abort the baby. Is it appropriate for you to perform an abortion in order to save the mother's life?”

Respondents inclined to deontological judgments typically oppose the abortion because it violates the unborn's right to life. In contrast, many subjects inclined to utilitarian judgments will consider the abortion as morally appropriate in this case because the loss of happiness to be experienced by the infant can be considered to be outweighed by the loss of happiness to be experienced by the mother (especially because the infant would grow up without a mother, thus experiencing a serious reduction in happiness).

Note that the differentiation between congruent and incongruent dilemmas is based on plausibility assumptions the process dissociation model makes in regards to certain moral judgments being consistent with utilitarian or deontological theoretical approaches to morality. To account for the individual variability in moral judgments, respondents are confronted with a series of congruent as well as incongruent dilemmas. Note also that the footbridge dilemma is an incongruent dilemma. The trolley dilemma is congruent in the sense that both moral inclinations take the same stance. However, in terms of above definition, the trolley dilemma is not congruent because both inclinations tend toward evaluating the action under consideration as appropriate (instead of inappropriate).

Process dissociation works with a parametrized model of judgment in moral dilemmas, according to which a participant's judgment in a dilemma variant may be consistent with either utilitarianism (U) or deontology (D), or with neither utilitarian (1—U) nor deontological (1—D) inclinations (Conway and Gawronski, 2013). Considering that the evaluation of a given behavior as unacceptable may be the result of two independent processes, the probability of judging a behavior as morally unacceptable in a congruent or incongruent dilemma is modeled by


(1) $$\begin{array}{l} \text{p }(\text{unacceptable | congruent})\;\equiv \;{\text{p}}_{\text{c}}\text{ = U }\\ \text{ + }[(\text{1 }-\text{ U})\;\times \text{ D}]\text{ and} \end{array}$$

(2) $$\begin{array}{l} \text{p }(\text{unacceptable | incongruent})\;\equiv \;{\text{p}}_{\text{i}}\\ \text{ = }(\text{1 }-\text{ U})\;\times \text{ D}. \end{array}$$

Confronting a participant with a set of congruent and incongruent dilemmas allows to measure the left-hand sides of Equations (1, 2). Therefore, the underlying parameters U and D can be calculated as


(3) $$\begin{array}{l} \text{U }=\;{\text{p}}_{\text{c}}\;-\;{\text{p}}_{\text{i}}\text{ and} \end{array}$$

(4) $$\begin{array}{l} \text{D }=\;{\text{p}}_{\text{i}}/\left(1\;-\;{\text{p}}_{\text{c}}+\;{\text{p}}_{\text{i}}\right). \end{array}$$

For instance, let us assume that a respondent is faced with five congruent and five incongruent dilemmas. Imagine that this respondent evaluates the behaviors in four of the five congruent dilemmas and in two of the five incongruent dilemmas as unacceptable. According to process dissociation and Equations (3, 4), the respondent's U is equal to 0.80 – 0.40 = 0.40 and the respondent's D is equal to 0.40/1-(0.80 – 0.40) = 2/3. Theoretically, U varies in the interval [−1.1], and higher values refer to a stronger inclination toward utilitarian judgments. D takes values between 0 and 1, whereby higher values indicate a stronger inclination toward deontological judgments.

Importantly, process dissociation does not invoke the assumption that U and D are opposing poles on a single continuum. That is, process dissociation does not assume that higher values of U necessarily go hand in hand with lower values of D. Instead, according to the model, U and D are independent parameters which jointly determine moral judgment in congruent as well as incongruent dilemmas. U and D are independent in the sense that a decision maker is characterized by both parameters and that the choice of a particular value of U does not a priori exclude the choice of a particular value of D. Of course, given empirical data on moral judgment, i.e., information on p(unacceptable | congruent) and p(unacceptable | incongruent), not all combinations of U and D do an equally good job in explaining the observations. This contrasts with the approach by Côté et al. (2013) who do not differentiate between a greater inclination toward utilitarian decisions and a smaller inclination toward deontological decisions. As already indicated, empirical research using process dissociation has uncovered that typically there is no strong and significant negative correlation between U and D (Conway and Gawronski, 2013; Conway et al., 2018; Fleischmann et al., 2019). In addition, empirical research using process dissociation has uncovered certain important predictors for moral judgment and more specifically U and D. For example, decisions may reflect processes such as concerns about following moral rules (Piazza and Landy, 2013), general inaction (Gawronski et al., 2017), or self-representation (Rom and Conway, 2018), as well as some aspects of emotional concern have also been shown to predict the utilitarian parameter, and some reasoning processes predict the deontology parameter (e.g., Reynolds and Conway, 2018; Byrd and Conway, 2019).

Note that the process dissociation model is based on the assumption that there are two qualitatively distinct types of moral judgment, and that the difference between congruent and incongruent moral dilemmas allows differentiating between them. However, any action described in a moral dilemma situation is situated in a context and is based on a number of assumptions, knowledge, beliefs and external constraints, which mediate the action. This could lead to potential ambiguities in regards to interpreting the moral dilemma situations and the associated decisions as having a clear and unequivocal moral meaning in accordance with either U or D logic. To be empirically tractable, these very abstract concepts have to be simplified. For the sake of simplicity, one can call the utilitarian parameter U “outcome-maximization decision tendencies” and the deontological parameter D “harm rejection-decision tendencies” to disentangle actual decision patterns people make from assumptions that these decision tendencies reflect abstract principles when they are merely consistent with those principles.

### 2.3 Hypotheses

Against this background, this paper aims at exploring the question how social class is associated with the inclinations toward utilitarian and deontological judgments if process dissociation is employed. Recall the original theoretical argument by Côté et al. (2013) which states that higher classes prefer utilitarian judgments and that this relationship is mediated by empathic concern. In formulating this argument, they assume utilitarian and deontological judgments as being two opposing poles on the same continuum. In contrast, we assume that inclinations toward utilitarianism or deontology are independent dimensions and apply the method of process dissociation to measure them as two different constructs on separate continua. We confront the respondents with five congruent and five incongruent dilemmas (see Appendix) and use their observed judgments to infer U and D on an individual level. This approach allows us to formulate two distinct hypotheses, expecting a negative association between social class and deontology and a positive one between social class and utilitarianism:

**H1**. *The higher the social class, the stronger the inclination toward utilitarian judgments*.
**H2**. *The higher the social class, the weaker the inclination toward deontological judgments*.

Note that in the pioneering study by Côté et al. (2013) H1 and H2 are essentially conflated and only the usage of process dissociation allows to discriminate between these two hypotheses.

Based on our theoretical arguments and former empirical work sketched above, our study also empirically investigates the extent to which class-based differences in moral judgment are driven by different potential mediating mechanisms, namely via empathic concern, thinking dispositions, and concern for moral identity. We challenge empathic concern as being the most powerful mediator and argue that thinking dispositions and moral identity could even be more important mediating mechanisms in the explanation of class-based differences in moral judgments. Thus, we put the following hypotheses regarding psychological mechanisms mediating the relationship between social class and moral judgment to the empirical test:

**H3**. *The relationship between social class and moral judgments is mediated by empathic concern*.
**H4**. *The relationship between social class and moral judgments is mediated by thinking dispositions*.
**H5**. *The relationship between social class and moral judgments is mediated by moral identity*.

Note that H3, H4, and H5 are structurally the same hypothesis, just varying different mediators. Nonetheless, to promote the transparency of our theoretical arguments and guide our empirical analyses we state these assertions as separate hypotheses. Note also that some of the theoretical arguments relating the mediating variables to moral inclinations are more plausible with respect to U or D. For instance, in reasoning that empathic concern is positively correlated with deontological judgments, Côté et al. (2013) effectively argue in favor of H2 and not necessarily of H1. Regardless of these details in theoretical argumentation, in this study we use the opportunity to check for mediation with respect to both utilitarianism and deontological judgments.

## 3 Study sample, methods, and variables

### 3.1 Study sample

Our study is an online survey implemented via respond, a German online access panel provider where people can voluntarily register to participate in opinion polls. Invitations to participate were sent to 13,591 persons randomly selected from the access panel. 3,465 subjects responded to the invitation link, resulting in an overall response rate of 25.5%. From these 3,465 respondents, 2,646 respondents provided their consent to participate in this study, passed a quality check question, and reached the end of the survey such that their data were included in our analysis. We employed listwise deletion (after imputing two variables, see below), leaving us with *N* = 2,515. 94 of 131 deletions occur because of missing values in D. These missing values occur because D is mathematically undefined if U equals 1. Our results regarding U are substantially identical if we do not delete these 94 cases. Data collection was conducted in February 2021 using SoSci Survey (https://www.soscisurvey.de) in German language.

### 3.2 Methods

Our measure for deontological (D) and utilitarian decision inclinations (U) is adapted from Conway and Gawronski (2013). Subjects were required to respond to five moral scenarios (*Abortion, Car Accident, Vaccine Policy, Animal Research*, and *Border Crossing*), each presented in an incongruent and congruent moral dilemma variation (see Appendix). A total of 10 dilemmas were translated into German and presented individually on separate screens in random order.

### 3.3 Variables

Inclinations toward utilitarian (U) and toward deontological judgments (D) were obtained by process dissociation. Recall that theoretically U can vary between −1 and 1. Empirically, U takes on values between −0.6 and 0.8 (*M* = 0.418, *SD* = 0.260). By construction D can only take values between 0 and 1; empirically the full range is observed (*M* = 0.480, *SD* = 0.328).

Our central independent variable is social class and it was constructed on the basis of income, education, and occupational prestige. Disposable income on the level of the household of the respondent was measured via an open question. Using information on the composition of the household we calculated equivalized disposable household income. We imputed values for 176 cases on the basis of a linear regression using the variables age, sex, migration, east, religiosity, political orientation, and education as predictors (*M* = 1, 483.096, *SD* = 783.662). To correct for implausible and extreme values, we constructed the variable “income group” which is an ordinal variable with values 1–4 based on the quartiles of equivalized disposable household income, with higher values indicating higher income.

Education was measured based on an instrument used in the European Social Survey that combines educational experience in schools and universities with vocational training and is appropriate for Germany's dual system in education (ESS, 2017). Our variable “education” differentiates between three groups, with higher values indicating higher forms of education (*M* = 1.870, *SD* = 0.795). The group with lowest education (1) consists of respondents who have obtained neither specialized vocational training nor a degree that allows to participate in higher education. The middle group (2) consists of respondents who received some specialized vocational training or have restricted access to higher education. The group with highest education (3) encompasses respondents who received intensive vocational training or completed a degree in higher education.

Occupational prestige was measured as follows. The respondents indicated their current job using a drop-down menu, which automatically converts the selected occupations into the ISCO-08 classification; from this, the ISCO-88 classification can be derived by means of a transformation table provided by the International Labor Organization (ILO, 2016). This allowed us to use a classification procedure (Stata-ado “ISKO”) developed by Ganzeboom and Treiman (1996, cf. also Ganzeboom and Treiman, 2011) and modified by Hendrickx (2004) to determine the “SIOPS” measure of occupational prestige (*M* = 42.916, *SD* = 13.122). The use of the drop-down menu resulted in a high number of missings; we imputed 1,191 missing values using a linear regression with the variables age, sex, migration, east, religiosity, political orientation, education, and income group as predictors.

Similar to Piff et al. (2010), we then standardized our variables income group, education, and SIOPS and calculated the arithmetic mean between these three variables (*M* = 0.004, *SD* = 0.711). Our measure of “social class” is the standardized version of this variable.

Regarding the potential mediators, i.e., cognitive styles, empathic concern, and moral identity, we relied on well-established scales from the literature (see Appendix for concrete items). The Cognitive Reflection Test consists of three questions each of which suggests an intuitive answer that is wrong (Frederick, 2005). The score in the Cognitive Reflection Test simply counts the number of correctly answered questions (*M* = 1.094, *SD* = 1.010). Faith in intuition (Epstein et al., 1996) was constructed as an additive index over 15 items using a scale from 1 to 7 (α = 0.890, *M* = 4.718, *SD* = 0.907). Empathic concern was measured using a German version (Grimm, 2015) of a subscale of Davis' Empathy Scale (Davis, 1980). It is an additive index over seven items using a scale from 1 to 8 (α = 0.799, *M* = 4.858, *SD* = 0.716). The Moral Identity Scale (Aquino and Reed, 2002) is an additive index over 10 items using a scale from 1 to 5 (α = 0.734, *M* = 2.952, *SD* = 0.565). Our variables “cognitive reflection,” “faith in intuition,” “empathic concern,” and “moral identity” are the standardized versions of the aforementioned indices.

In our multivariate analyses we make use of a number of control variables. “Age” is a metric variable counting a respondent's age in years (*M* = 49.142, *SD* = 15.993). “Sex” is a dummy taking value 1 if the respondent is a female (*M* = 0.519). “East” is a dummy indicating whether the respondent currently lives in the eastern part of Germany (*M* = 0.216). “Urban” is a dummy which takes value 1 if the respondent lives in a big city or a suburb of a big city (*M* = 0.405). “Migration” is a dummy indicating whether at least one of the respondent's parents was not born in Germany (*M* = 0.136). “Couple” is a dummy which takes value 1 if the respondent is married and living together with his or her spouse (*M* = 0.492). “Religiosity” is a (pseudo-)metric variable measuring on a scale from 1 (never) to 7 (every day) how frequently the respondent is participating in religious ceremonies (*M* = 1.773, *SD* = 1.141). “Political orientation” is a metric variable measuring on a scale from 1 (left) to 11 (right) the general political orientation of the respondent (*M* = 5.011, *SD* = 1.915). All metric control variables (age, religiosity, and political orientation) were standardized before entered into the multivariate analyses.

## 4 Empirical results

### 4.1 Bivariate relationships

We find a small but significant negative correlation between U and D (*corr* = −0.168, *p* < 0.000). Regarding the strength of the correlation, this finding is in line with results from the studies by Conway and Gawronski (2013) and Conway et al. (2018). The fact that the negative correlation is statistically significant is related to our rather sizeable sample. Still, our findings back the fundamental idea that utilitarian and deontological decision inclinations should not be thought of as opposing poles on a continuum.

**Figure 1:**
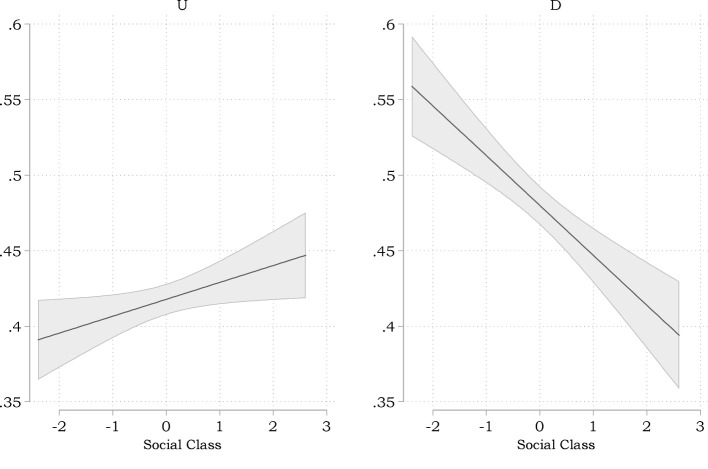
Correlations between social class and moral inclinations (predictions based on bivariate regressions. Gray areas indicate 95% confidence intervals).

Figure 1 depicts how social class correlates with both moral inclinations. We find a very weak yet statistically significant positive correlation between social class and utilitarian decision inclinations (*corr* = 0.043, *p* < 0.031. Furthermore, upper-class respondents are somewhat less inclined to deontological judgments (*corr* = −0.101, *p* < 0.000).

To gain a first insight into potential mediation mechanisms between social class and moral inclinations, Table 1 displays correlation coefficients between social class as well as U and D and the potentially mediating variables cognitive reflection, faith in intuition, empathic concern, and moral identity. Social classes do differ in thinking dispositions, in particular as measured by the Cognitive Reflection Test, but differ only slightly in empathic concern and the importance they place on having a moral identity. While the inclination toward utilitarian decisions is noticeably associated with the tendency toward cognitive reflection and a bit with the importance respondents place on having a moral identity, the correlations with faith in intuition and empathic concern are rather weak. Interestingly, the inclination toward deontological judgments is not correlated with any of the potential mediators.

**Table 1:** Correlations between social class, U and D and the potential mediators (*P*-values in parentheses).

	**Social class**	**U**	**D**
Cognitive reflection	0.261^***^	0.171^***^	−0.022
	[0.000]	[0.000]	[0.279]
Faith in intuition	−0.109^***^	−0.050^**^	0.013
	[0.000]	[0.013]	[0.515]
Empathic concern	−0.076^**^	−0.076^**^	−0.027
	[0.001]	[0.001]	[0.180]
Moral identity	0.034^+^	−0.126^***^	0.000
	[0.084]	[0.000]	[0.993]

^+^p < 0.1, ^*^p < 0.05, ^**^p < 0.01, ^***^p < 0.001.

Table 2 provides pairwise correlations between the potential mediating variables. We find moderate positive correlations between faith in intuition, empathic concern, and moral identity. While there is a moderate negative correlation between cognitive reflection and faith in intuition, the negative correlations between cognitive reflection and empathic concern as well as between cognitive reflection and moral identity are rather weak.

**Table 2:** Correlations between the potential mediators (*P*-values in parentheses).

	**Cognitive reflection**	**Faith in intuition**	**Empathic concern**
Cognitive reflection	X	−0.161^***^	−0.034^+^
		[0.000]	[0.087]
Faith in intuition	X	X	0.367^***^
			[0.000]
Empathic concern	X	X	X
Moral identity	−0.078^***^	0.304^***^	0.345^***^
	[0.000]	[0.000]	[0.000]

^+^p < 0.1, ^*^p < 0.05, ^**^p < 0.01, ^***^p < 0.001.

These findings suggest that class-based differences in the inclination toward utilitarianism are potentially due to differences in the tendency toward cognitive reflection and, to a smaller extent, due to differences in empathic concern. Neither cognitive styles nor empathic concern and the importance of a positive moral identity seem to explain the negative relationship between social class and the inclination toward deontology. The following subsection checks whether these bivariate findings are robust in multivariate analyses.

### 4.2 Multivariate analyses

Tables 3, 4 display a number of linear regression models which allow to replicate the results from the bivariate analyses in a multivariate context. Besides social class and the potentially mediating variables cognitive reflection, faith in intuition, empathic concern, and moral identity these models include standard control variables referring to basic sociodemographic characteristics as well as religiosity and general political orientation. Models 1–4 in Table 3 refer to utilitarianism (U), whereas models 5–8 in Table 4 refer to deontology (D).

**Table 3:** Multiple linear regressions using U as dependent variable (All variables are either dummies or standardized. Standard errors in parentheses).

	**Model 1**	**Model 2**	**Model 3**	**Model 4**
Age	0.018^**^	0.023^***^	0.013^*^	0.018^**^
	(0.006)	(0.005)	(0.006)	(0.006)
Sex	−0.024^*^	−0.010	−0.026^*^	−0.015
	(0.011)	(0.011)	(0.011)	(0.011)
Migration	−0.029^+^	−0.026^+^	−0.029^+^	−0.026^+^
	(0.015)	(0.015)	(0.015)	(0.015)
East	−0.017	−0.008	0.014	−0.007
	(0.013)	(0.013)	(0.013)	(0.013)
Urban	−0.022^*^	−0.020^+^	−0.020^+^	−0.019^+^
	(0.011)	(0.011)	(0.011)	(0.011)
Couple	0.012	0.015	0.013	0.015
	(0.011)	(0.011)	(0.011)	(0.011)
Religiosity	−0.013^*^	−0.009^+^	−0.007	−0.005
	(0.005)	(0.005)	(0.005)	(0.005)
Political orientation	−0.000	−0.001	−0.000	−0.002
	(0.005)	(0.005)	(0.005)	(0.005)
Social class	0.013^*^	0.001	0.012^*^	0.002
	(0.005)	(0.005)	(0.005)	(0.005)
Cognitive reflection		0.045^***^		0.044^***^
		(0.005)		(0.005)
Faith in intuition		−0.006		0.005
		(0.005)		(0.006)
Empathic concern			−0.010^+^	−0.012^*^
			(0.006)	(0.006)
Moral identity			−0.025^***^	−0.022^***^
			(0.006)	(0.006)
Constant	0.441^***^	0.429^***^	0.440^***^	0.431^***^
	(0.011)	(0.011)	(0.011)	(0.011)
*R* ^2^	0.019	0.047	0.031	0.056
*N*	2, 515	2, 515	2, 515	2, 515

^+^p < 0.1, ^*^p < 0.05, ^**^p < 0.01, ^***^p < 0.001.

**Table 4:** Multiple linear regressions using D as dependent variable (all variables are either dummies or standardized. Standard errors in parentheses).

	**Model 5**	**Model 6**	**Model 7**	**Model 8**
Age	0.019^**^	0.020^**^	0.019^**^	0.020^**^
	(0.007)	(0.007)	(0.007)	(0.007)
Sex	0.092^***^	0.095^***^	0.091^***^	0.094^***^
	(0.013)	(0.013)	(0.013)	(0.013)
Migration	0.032^+^	0.033^+^	0.031	0.032^+^
	(0.019)	(0.019)	(0.019)	(0.019)
East	−0.032^*^	−0.030^+^	−0.033^*^	−0.031^+^
	(0.016)	(0.016)	(0.016)	(0.016)
Urban	−0.009	−0.009	−0.009	−0.009
	(0.013)	(0.013)	(0.013)	(0.013)
Couple	−0.013	−0.012	−0.013	−0.013
	(0.013)	(0.013)	(0.013)	(0.013)
Religiosity	0.012^+^	0.013^*^	0.012^+^	0.012^+^
	(0.007)	(0.007)	(0.007)	(0.007)
Political orientation	−0.024^***^	−0.024^***^	−0.023^***^	−0.023^***^
	(0.007)	(0.007)	(0.007)	(0.007)
Social class	−0.030^***^	−0.033^***^	−0.031^***^	−0.033^***^
	(0.006)	(0.007)	(0.007)	(0.007)
Cognitive reflection		0.009		0.010
		(0.007)		(0.007)
Faith in intuition		−0.001		0.001
		(0.007)		(0.007)
Empathic concern			−0.010	−0.010
			(0.007)	(0.007)
Moral identity			0.004	0.004
			(0.007)	(0.007)
Constant	0.455^***^	0.443^***^	0.445^***^	0.444^***^
	(0.014)	(0.014)	(0.014)	(0.014)
*R* ^2^	0.042	0.042	0.042	0.043
*N*	2,515	2,515	2,515	2,515

^+^p < 0.1, ^*^p < 0.05, ^**^p < 0.01, ^***^p < 0.001.

Model 1 depicts a very small effect of social class on utilitarian judgments. On average, an increase in social class by one standard deviation leads to an increase in the inclination toward utilitarianism by 0.01. This coefficient is significant at the 5% level. Model 2 reveals that this very slight difference in utilitarian decision inclinations between classes stems from differences in cognitive styles. While it is true that a higher empathic concern and a stronger focus on a positive moral identity diminish the inclination toward utilitarian judgments, Models 3 and 4 suggest that both variables do not mediate the class effect on utilitarian decisions.

**Figure 2:**
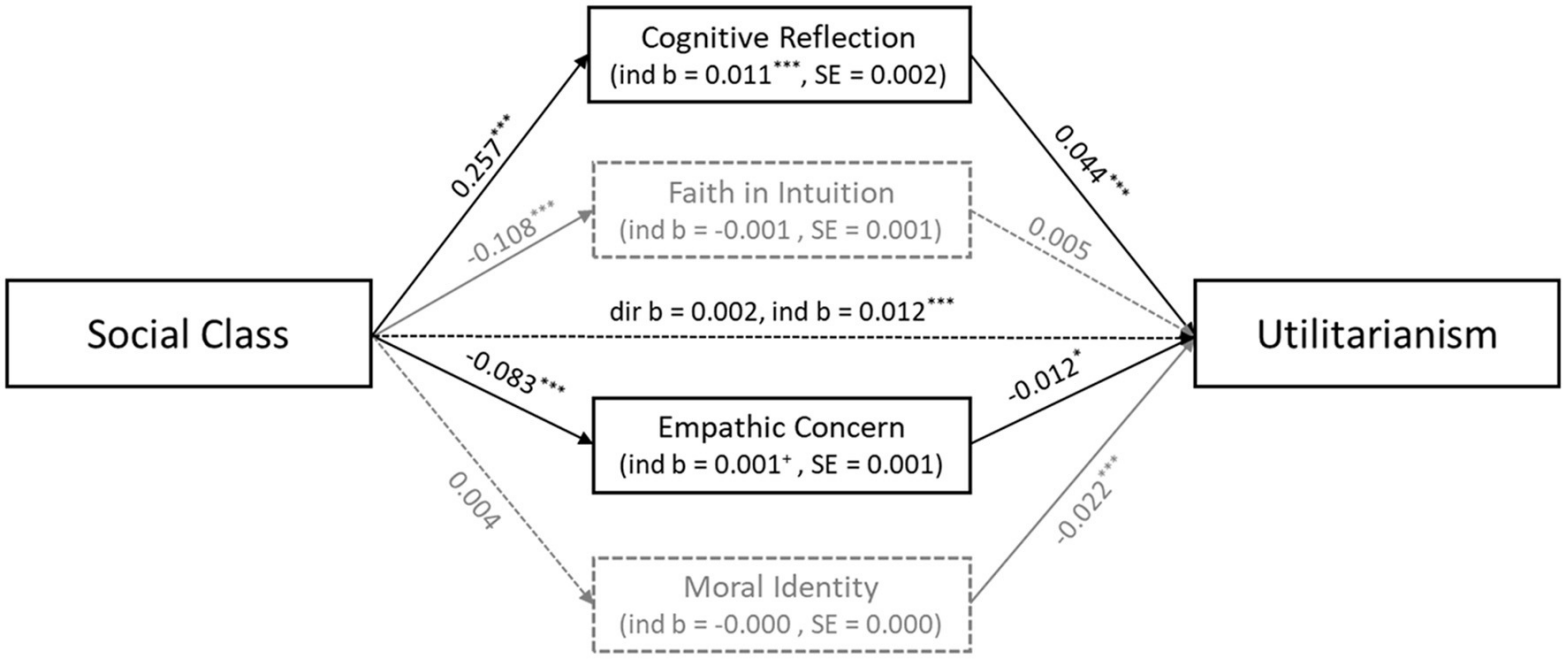
Direct and indirect effects of social class on U (Bold lines indicate significant direct effects and dotted lines indicate non-significant direct effects. Significant indirect effects in black, non-significant in gray). ^+^p < 0.1, ^*^p < 0.05, ^**^p < 0.01, ^***^p < 0.001.

Figure 2 depicts the results of a mediation analysis to further clarify the direct and indirect effects of social class on the inclination toward utilitarianism. The (simultaneous) mediation analysis was conducted with Stata. Our standard control variables were used in all regressions involved. Standard errors of indirect effects were bootstrapped (1,000 samples). Due to rounding errors, direct and indirect effects do not necessarily add up to total effects. The total effect of social class on the inclination toward utilitarian judgments is obtained from a regression that uses social class and the control variables but not the potential mediators as independent variables (Model 1 in Table 3); the total effect of social class on U equals 0.013 (*p* < 0.012). To get an estimate for the direct effect of social class, we regress U on social class, the control variables, and all of the potential mediators (Model 4 in Table 3) and obtain a coefficient of 0.002 (*p* < 0.762). In a linear regression setup, the direct and indirect effect of a variable necessarily add up to its total effect. Hence, the indirect effect of social class on the inclination toward utilitarian judgments which is transmitted via the potential mediators equals 0.012 (*p* < 0.000).

Mediation analysis allows to determine the contribution of each of the potential mediators to the indirect effect of social class. To obtain these estimates, we proceed in two steps. First, we regress each of the potential mediators on social class and the control variables. The coefficients of social class from these regressions are depicted next to the arrows on the left-hand side of Figure 2. Second, we determine the coefficients of the potential mediators in a model which regresses U on social class, the control variables as well as the potential mediators (Model 4 in Table 3); these estimates are depicted next to the arrows on the right-hand side in Figure 2. The indirect effect of social class which flows via a specific potential mediator simply equals the product of the estimates along the corresponding two arrows.

The results of our mediation analysis can be summarized as follows: The indirect effect of social class on the inclination toward utilitarian judgments flows via two significant channels. First, upper-class actors score higher in the Cognitive Reflection Test (*b* = 0.257, *p* < 0.000) and higher scores in the Cognitive Reflection Test are associated with higher values in U (*b* = 0.044, *p* < 0.000). The indirect effect via this mechanism equals 0.011 and is highly significant (*p* < 0.000). Second, upper-class individuals show less empathic concern (*b* = −0.083, *p* < 0.000) and empathic concern is negatively correlated with the inclination toward utilitarianism (*b* = −0.012, *p* < 0.034). This indirect effect equals 0.001 and is significant at the 10% level (*p* < 0.083). The indirect effects via faith in intuition and moral identity are not significant. The difference between the indirect effect of cognitive reflection and the indirect effect of empathic concern equals 0.010 with a (bootstrapped) standard error of 0.002 and is statistically different from zero (*p* < 0.000). Against this background, it is fair to say that the effect of social class on the inclination toward utilitarian judgments is mediated to a great extent by cognitive reflection and to some extent by empathic concern.

Model 5 reveals that social classes do differ more substantially with respect to their inclination to deontological judgments. An increase in social class by one standard deviation goes hand in hand with a decrease in D by 0.03. The coefficient is significant at the 0.1% level. Since U varies theoretically between −1 and 1, while D only varies between 0 and 1, we consider the difference between the −0.03 coefficient in Model 4 and the 0.01 coefficient in Model 1 to be sizeable. As Models 5–8 demonstrate, the differences in the inclination toward deontological judgments between social classes are neither explained by cognitive styles nor by empathic concern and moral identity.

While the impact of social class on moral inclinations seems very modest, considering the coefficients of the control variables as well as the model fits puts that impression into context. Apparently, only a tiny fraction of the utilitarian and deontological decision inclinations is explained by our explanatory and control variables (*R*^2^ ≤ 0.056 and *R*^2^ ≤ 0.043, respectively). Social class has a comparable (with respect to U) or even slightly stronger (with respect to D) effect than both religiosity and political orientation. With the exception of gender, social class is the strongest predictor of the inclination toward deontological decisions among all variables under consideration.

Hence our multivariate analyses back our preliminary conclusions from the bivariate analyses: Social classes do differ a little bit in their inclination toward utilitarian judgments and a bit more in their inclination toward deontological decisions. While class-based differences in utilitarian decision inclinations are explained by differences in cognitive styles, neither cognitive styles nor empathic concern and a concern for a moral identity do explain class-based differences in the inclination toward deontological judgments. Keeping in mind that variations in both U and D are hard to explain by all of the explanatory variables under consideration, social class appears to be a rather important predictor for the inclination toward deontological decisions.

### 4.3 Additional analyses

Hitherto, we relied on process dissociation to measure U and D and test whether there are any differences between classes with respect to these measures of moral inclinations. In this subsection, we stick more closely to the approach by Côté et al. (2013) and employ a different analytical strategy to test the central hypothesis that actors with a higher social class are more inclined to utilitarian judgments.

Recall, Côté et al. (2013) confront their subjects with the trolley dilemma and the footbridge dilemma. The trolley dilemma is congruent in the sense of not exhibiting a hard clash between utilitarianism and deontological principles (Greene, 2013). Typically, actors inclined to utilitarian as well as actors inclined to deontological judgments find the action under consideration morally appropriate. The footbridge dilemma is an incongruent dilemma, since people who make utilitarian decisions tend to judge the action as morally appropriate, while actors inclined to decisions consistent with deontological principles evaluate it as morally inappropriate. Côté et al. (2013) demonstrate that social class has a stronger effect on judging the behavior morally appropriate in the incongruent footbridge dilemma than in the congruent trolley dilemma and interpret this finding to indicate that upper-class actors are more inclined to decisions consistent with utilitarian principles than lower-class actors.

As indicated, our measures of U and D are based on five pairs of dilemmas, each pair consisting of a congruent and incongruent version. This provides us with the opportunity to conduct five replication tests of this interaction effect between type of dilemma (congruent vs. incongruent) and social class, one for each pair of dilemmas.

**Figure 3:**
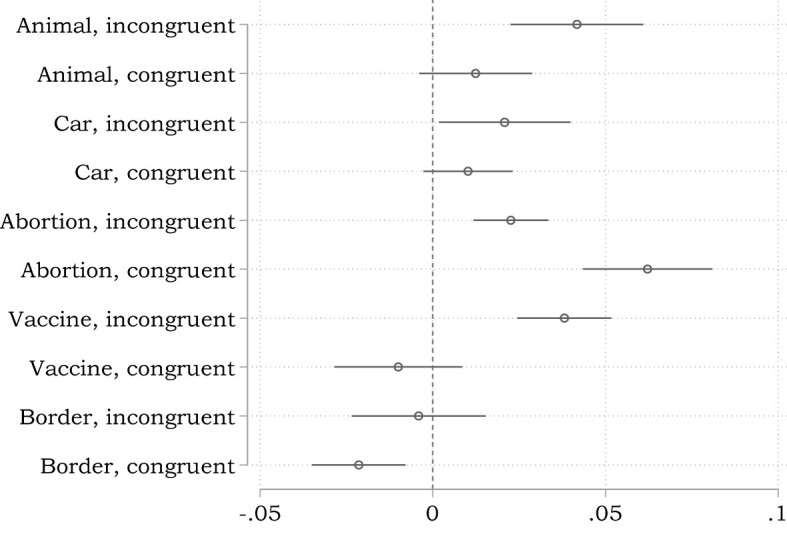
Effect of social class on the probability of evaluating the action under consideration as morally appropriate in congruent and incongruent dilemmas (Coefficients from linear probability models, estimated separately for each of the 10 dilemmas. The models encompass our standard control variables, but do not encompass the potential mediators cognitive reflection, faith in intuition, empathic concern, and moral identity).

Figure 3 presents the results of these five replication tests. The figure depicts the coefficient of social class from 10 separate models (one for each dilemma) in which the probability of evaluating the action under consideration as morally appropriate is regressed on all of our control variables and social class. Following the recent literature on non-linear probability models and the problems of these models in dealing with interaction effects, we rely on the linear probability model (Ai and Norton, 2003; Breen et al., 2018). As a consequence, the depicted coefficients can be interpreted as an approximation of average marginal effects. For instance: In the congruent version of the abortion dilemma, an increase in social class by one standard deviation leads to an ~6.2% increase in the probability of evaluating the abortion as morally appropriate.

We find mixed evidence with respect to the interaction effect between type of dilemma and social class. Descriptively, social class has a stronger effect in the incongruent than in the congruent version in four of the five dilemmas (i.e., animal, car, vaccine, and border). Additional analyses with an explicit interaction term reveal that this interaction only obtains significance in the animal and the vaccine dilemma (animal: *coef* = 0.028, *p* < 0.006; car: *coef* = 0.009, *p* < 0.425; vaccine: *coef* = 0.041, *p* < 0.000; border: *coef* = 0.017, *p* < 0.110). Moreover, in the abortion dilemma, social class even has a significantly lower effect in the incongruent than in the congruent version (*coef* = −0.039, *p* < 0.000). Additional unreported analyses reveal that our findings are robust, whether we include control variables or not and whether we rely on linear or non-linear probability models.

Following the argument by Côté et al. (2013) and also in line with our previously reported results using process dissociation, we interpret these findings to indicate that there is a slight tendency toward a greater inclination toward utilitarian judgments among subjects with a higher social class.

**Figure 4:**
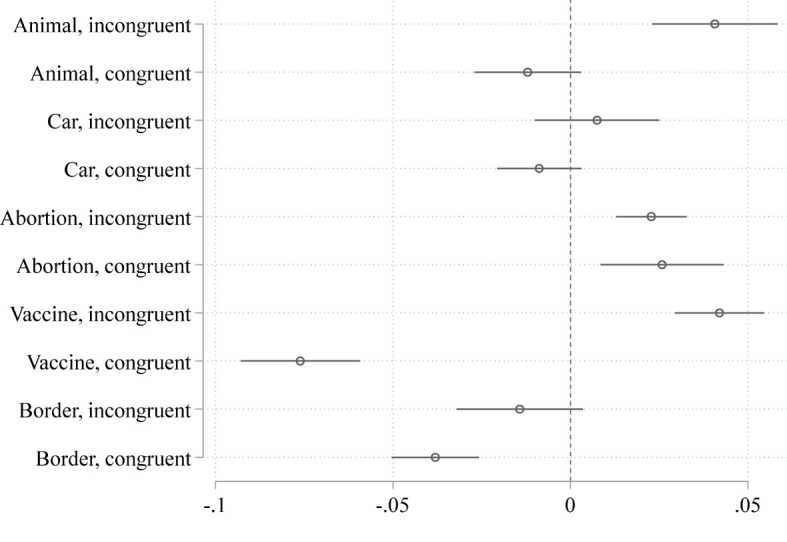
Effect of the CRT score on the probability of evaluating the action under consideration as morally appropriate in congruent and incongruent dilemmas (Coefficients from linear probability models, estimated separately for each of the 10 dilemmas. The models encompass our standard control variables, but do not encompass social class and the potential mediators faith in intuition, empathic concern, and moral identity).

It is also worthwhile to check whether our findings regarding the mediating role of cognitive styles can be replicated using this alternative analytical strategy. In a first step, we check how differences in the CRT score affect moral judgment in congruent as well as incongruent dilemmas. Figure 4 is analogous to Figure 3; that is, we depict the coefficient of the CRT score from 10 separate models (one for each dilemma) in which the probability of evaluating the action under consideration as morally appropriate is regressed on all of our control variables and the CRT score.

We find that with the sole exception of the abortion scenario, the CRT score has a greater effect in the incongruent than in the congruent dilemmas. The fact that the pattern visible in Figure 4 is similar but more pronounced than the pattern depicted in Figure 3 suggests that class-based differences in moral judgment might be due to class-based differences in cognitive styles.

**Figure 5:**
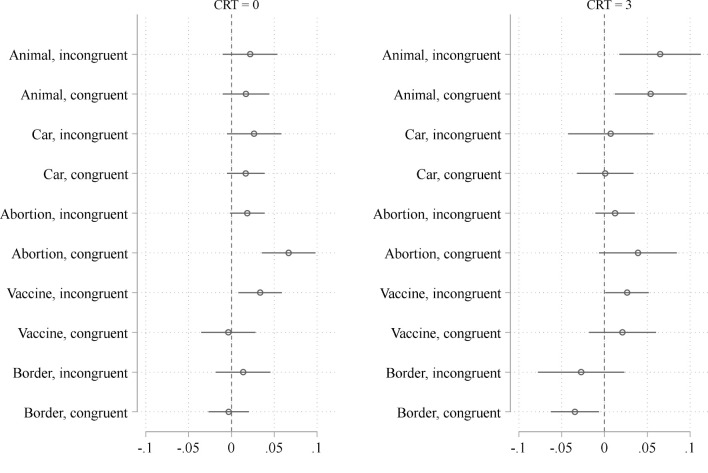
Effect of social class on the probability of evaluating the action under consideration as morally appropriate in congruent and incongruent dilemmas stratified for cognitive style (Coefficients from linear probability models, estimated separately for each of the 10 dilemmas. The models encompass our standard control variables, but do not encompass the potential mediators cognitive reflection, faith in intuition, empathic concern, and moral identity).

To further investigate the potential mediating role of cognitive style, we stratify our sample according to the CRT score and redo our analyses regarding the effect of social class. More specifically, Figure 5 is based on the same type of models used in the construction of Figure 3, but estimated separately for those subjects who obtain a score of 0 on the CRT (*N* = 1,028) on the one hand, and those subjects, who obtain a score of 3 on the CRT (*N* = 384) on the other hand. If the observed differences in Figure 3 are largely due to class-based differences in cognitive style, we should find comparatively small differences in the effect of social class between the congruent and incongruent versions of the dilemmas.

Figure 5 supports the idea that cognitive style mediates the effects of social class on moral judgment. That is, congruent and incongruent dilemmas do not differ much in the extent to which social class influences moral judgment given that we control for the CRT score. Among highly reflected subjects, the effect of social class does not differ at all between the incongruent and the congruent versions of the dilemmas. Among very intuitive subjects, only two of five scenarios exhibit a significant difference: The abortion scenario, which is an anomaly in the first place, because the effect of social class is actually greater in the congruent than in the incongruent version, contrary to theoretical expectations (*coef* = −0.046, *p* < 0.007). In addition, we find a relatively pronounced and significant difference in effect strength in the vaccine scenario (*coef* = 0.032, *p* < 0.08).

## 5 Discussion

In the following, we will summarize the key findings of our study regarding class-related differences in moral judgment, as well as with respect to the influence of the mediators examined. Further, we relate our results and methodology to the study by Côté et al. (2013). Finally, the limitations of the present study will be discussed and conclusions for upcoming studies will be derived.

Starting with the main findings of our study, we note that that class-based differences in deontological decision inclinations are more pronounced than class-based differences in utilitarian decision tendencies. In addition, we find that the effect of social class on utilitarian judgments is mediated by cognitive reflection and, to a much smaller extent, by empathic concern. Neither cognitive styles nor empathic concern or moral identity explain the negative relationship between social class and the inclinations toward deontological judgments.

In comparison to Côté et al. (2013), our study paints a more nuanced picture regarding the relationship between social class and the inclinations toward utilitarian and deontological decisions. While it is true that upper-class actors do indeed show a greater inclination toward utilitarian decisions than lower-class actors, the total effect of social class is rather small. The fact that our results differ from that of Côté et al. (2013) is certainly related to our different methodology; while in the study by Côté et al. (2013) utilitarian and deontological judgments were conceptualized as opposing poles on a single continuum, we employ process dissociation to measure the inclinations toward utilitarian and deontological decisions as independent and orthogonal concepts. Because the methodological approach of Côté et al. (2013) conflates a higher tendency toward utilitarian judgments with a lower tendency toward deontological judgments, the former study necessarily overestimated class-based differences in utilitarian decision tendencies and underestimated class-based differences in deontological decision inclinations. While it is true that differences in empathic concern between the classes explain partly class-based differences regarding utilitarian judgments, our study demonstrates that differences in the tendency toward cognitive reflection are considerably more important.

Our approach is similar to the study by Fleischmann et al. (2019), who also employ process dissociation to study the differential effects of power on moral judgment. Their study also makes use of the recently developed moral orientation scale to assess four thinking styles which relate to cognitive reflection and empathy. Their central finding is that the total effect of power on U and D might be null because power influences simultaneously thinking styles that enhance or diminish the inclinations toward utilitarian and deontological judgments. Against the background of these findings and the fact that power and class are conceptually distinct but empirically related concepts (Kraus et al., 2010; Piff et al., 2010), future research on the class-based differences in moral reasoning could greatly benefit from employing the moral orientation scale.

The fact that in our study empathic concern does play a much smaller role in explaining class-based differences in moral judgment might be related to the fact that we simply measured empathy in contrast to Côté et al. (2013) who manipulated it experimentally. In addition, their experimental manipulation involved empathy regarding concrete persons, i.e., losers in an allocation task, whereas our measure of empathy is more abstract. Future research should study the relative strength of cognitive styles as well as empathic concern using designs in which both cognitive styles and empathic concern are included as potential mediators and manipulated experimentally (Spencer et al., 2005). For example, to examine whether cognitive styles are a more powerful mediator than empathy, both variables could be experimentally manipulated in a 2 x 2 ANOVA design to examine the relative strengths of the effects on moral judgments.

Turning to the limitations of the present study, we would like to point out that since this is not a cross-cultural study, the results cannot easily be generalized to other populations and cultures. For instance, Nisbett et al. (2001) as well as Norenzayan et al. (2002) show that Westerners prefer analytic thinking styles in contrast to Easterners. Therefore, cultural factors may play a role, when it comes to understand class-related differences in utilitarian and deontological moral judgment mediated by cognitive styles. Furthermore, it can be assumed that different moral decision-making patterns exist due to different moral values, which vary, for example, between individualistic and collectivistic cultures (Rhim et al., 2020).

Another important limitation of the present study comes from the reliance on mediation analysis and its limited potential regarding causal identification. From the point of view of the modern literature on causality (Morgan and Winship, 2015), which combines the potential outcome framework (Imbens and Rubin, 2015) with Pearl's conception of causality (Pearl, 2000), mediation analysis is susceptible to endogenous selection bias (e.g., Elwert, 2013, p. 264). Put briefly, in estimating the direct effect of social class on moral judgment, we control for thinking dispositions. Any unobserved confounder between thinking dispositions and U or D confounds this estimate because the mediator figures as a collider on a non-causal path. While many of the most obvious potential confounders between thinking dispositions and moral inclinations are either captured by our social class variable or controlled for, such as age, gender, and religiosity, other potential confounders such as intelligence, (sub)cultural background, and personality traits are unobserved and therefore neglected. Despite the conceptual advantages of causal mediation analysis, which provides clearer definitions of direct and indirect effects and uses advanced graphical models to outline causal relationships, it cannot resolve the issue of endogenous selection bias (Pearl et al., 2016; Byeon and Lee, 2023). The method's stringent conditions for causal identification, which exclude scenarios with unobserved confounders, indicate that both traditional and causal mediation analyses are limited in this respect. Hence, while fairly common in the study of moral judgment (e.g., Fleischmann et al., 2019), results from mediation analyses such as the one conducted in this paper must be taken with a grain of salt when it comes to interpreting them from a causal point of view. Note that our claims regarding mediation are not only backed by a formal mediation analysis but also supported by two additional analytical strategies, i.e., nested regressions and comparisons between pairs of congruent and incongruent dilemmas.

A further limitation of the present study concerns the implementation of process dissociation. While process dissociation is considered a viable analytical tool to test ideas from the dual-process perspective (Payne and Cameron, 2014) and has been proven fruitful in applied research on moral judgment (Conway and Gawronski, 2013; Conway et al., 2018), the method also has considerable inherent limitations. One of the most important drawbacks of our application of process dissociation relates to the simplistic measurement of moral inclinations. It can be reasonably doubted that abstract moral principles do as neatly as the method suggests break down into overt moral judgment in the concrete moral dilemmas featured in our study. This is especially troublesome with respect to deontology; in essence, our implementation of process dissociation focuses on just one, albeit important aspect of deontology, i.e., the principle that doing actively harm is morally unacceptable (Kant, 1797).

Against this background, it is reassuring that we were able to replicate one of our major findings, i.e., that class-based differences in moral judgment are to a large extent due to class-based differences in cognitive styles, using an alternative analytical strategy that does not make use of process dissociation and instead directly compares moral judgment in incongruent and congruent dilemmas. In interpreting this finding, it is important to note that the alternative method, which does not make use of process dissociation, does not necessarily support the claim that cognitive styles do mediate class-based differences in the inclination toward utilitarian judgments. A plausible and alternative interpretation of these findings is that in comparison to congruent dilemmas, incongruent dilemmas involve greater moral ambiguity which warrant some type of reflective cost-benefit analysis to be resolved and the ability to engage into this type of analysis is socially patterned.

Another limitation of the current study is that we missed out on measuring an important potential mediator of the relationship between social class and moral judgment. That is, Kraus et al. (2012) argue on theoretical grounds and cite evidence for that higher-class actors generally put a greater emphasis on an agentic self-concept, whereas lower-class actors tend more toward a communal self-concept. Since a more agentic conception of one's own self goes hand in hand with a greater belief into the capacity to control outcomes and the confidence to make unique choices (Markus and Kitayama, 2010), this factor might partly explain observed differences in moral judgment, in particular in the more ambiguous and conflicted incongruent dilemmas.

Finally, we consider it the greatest limitation of the current study that our analyses are purely observational. While objective class is difficult or even impossible to be manipulated experimentally, there are well-established techniques in psychological research of manipulating subjectively perceived social class and status (Piff et al., 2012). It is an important task for future research to implement such manipulations in appropriate designs to learn more about causal effects of social class on moral judgment.

## Data availability statement

The raw data supporting the conclusions of this article will be made available by the authors, without undue reservation.

## Ethics statement

Ethical approval was not required for the studies involving humans because the procedures used in this study adhere to the tenets of the Declaration of Helsinki and APA standards. The studies were conducted in accordance with the local legislation and institutional requirements. The participants provided their written informed consent to participate in this study.

## Author contributions

AT: Writing – original draft, Writing – review & editing. FH: Writing – original draft, Writing – review & editing. IK: Writing – original draft, Writing – review & editing.

## Footnotes

^1^While we employ mediation analysis in this paper because the theoretical arguments put forward in the literature on class-based differences in moral judgment suggest this type of analysis, we acknowledge the inherent limitations of this method in identifying causal effects due to endogenous selection bias. The reader is referred to Section 5 for more elaborate reflections on this issue.
