# Supplementary material for: Social class and moral judgment: a process dissociation perspective
Source: Front Sociol. 2024 Apr 30;9:1391214. doi: 10.3389/fsoc.2024.1391214 (PMC11092982; doi:10.3389/fsoc.2024.1391214)
Supplement: Supplementary file 1 [file Data_Sheet_1.docx]

Appendix

Social Class and Moral Judgment:

A Process Dissociation Perspective

Contents

A0 Introduction p. 2

A1 Moral Dilemmas p. 2

A2 Potential Mediators p. 6

A3 References p. 9

A0 Introduction

This supplement is structured as follows: First, we provide information on the moral dilemmas used to measure inclinations towards utilitarianism and deontology by process dissociation. Second, we present the implementation of moral dilemmas. In the last section we give additional information about the measure of the potential mediators.

A1 Moral Dilemmas

Our measure of deontological (D) and utilitarian inclinations (U) was adapted from Conway & Gawronski (2013). Participants were asked to respond to five moral scenarios (*Abortion, Car Accident, Vaccine Policy, Animal Research, Border Crossing*), each being presented in a congruent and incongruent moral dilemma variant. Incongruent dilemmas were designed to test deontological choices against utilitarian choices. In an incongruent dilemma, utilitarianism evaluates the behavior under consideration as morally acceptable, whereas deontology evaluates the behavior as unacceptable. For instance, in the incongruent variant of the *border crossing dilemma* participants are asked to imagine they are a soldier guarding a border checkpoint. Then they have to judge the appropriateness of shooting and killing a man, which they suspect to bomb the checkpoint killing all the soldiers inside. In the incongruent dilemmas the consequences of harmful actions were described as beneficial compared to the harm of the action per se.

Unlike the incongruent dilemmas, in the congruent version a harmful action can prevent an undesirable event, but the harmful action per se causes more harm than public good. In a congruent dilemma, both utilitarianism and deontology evaluate the behavior under consideration as morally unacceptable. For example, in the congruent variant of the *border crossing dilemma* respondents again assume the role of a soldier and then have to judge whether they believe that it is appropriate or not to shoot and kill a man, which they suspect to cross the border in order to work illegally inside the participants country. Killing the man approaching the border in this case causes more harm than overall well-being and is considered unacceptable by both deontological and utilitarian criteria. In each dilemma, the subjects were described as acting protagonists, who had to decide whether the performance of a certain harmful action would be *appropriate* or *inappropriate*. In total, ten dilemmas were translated into German and presented individually on separate screens in a random order:

**Abortion**

*Incongruent Dilemma Variant*

“You are a surgeon. A young woman you know becomes pregnant, but her body reacts in an unusual fashion. She develops a severe case of preeclampsia, a dangerous syndrome that leads to rapid increases in blood pressure. The only treatment is to deliver the baby. Unless the baby is delivered soon, the mother will die. However, the baby is too young to survive on its own. If it is delivered, it will die. So, although it is very difficult for her, the mother asks you to abort the baby. Is it appropriate for you to perform an abortion in order to save the mother’s life?”

*Congruent Dilemma Variant*

“You are a surgeon. A young woman you know becomes pregnant, but she is not yet ready for children. She has not finished high school, has no income, and was abandoned by the father. If she has the baby now, she will be stuck as a single mother on welfare for the rest of her life. This will make things very hard on her and the baby. She thinks that it would be smarter to wait and have children later. So, although it is very difficult for her, she asks you to abort the baby. Is it appropriate for you to perform an abortion in order to let the mother live a better life?”

**Vaccine Policy**

*Incongruent* *Dilemma Variant*

“You are a doctor in a health clinic overrun by patients with a serious disease. You just received a shipment of drugs that can cure the disease but the drugs have their own severe side effects. If you administer the drugs to your patients, a small number will die from the side effects but most will live. If you do not, most will die from the disease. Is it appropriate for you to administer the drug to your patients?”

*Congruent Dilemma Variant*

“You are a doctor in a health clinic overrun by patients with the latest flu virus. You just received a shipment of drugs that can cure the flu but the drugs have their own severe side-effects. If you administer the drugs to your patients, a small number will die from the side effects but most will live. If you do not, most will continue to suffer from the effects of the flu virus for some time. Is it appropriate for you to administer the drug to your patients?”

**Border Crossing**

*Incongruent Dilemma Variant*

“You are a soldier guarding a border checkpoint between your nation and one troubled by insurgent violence. You notice a young man in a cheap car approaching the checkpoint with a

determined look on his face. You suspect he means to bomb the checkpoint, killing all the soldiers inside. He is rapidly approaching your station. Is it appropriate for you to shoot and kill the approaching man?”

*Congruent Dilemma Variant*

“You are a soldier guarding a border checkpoint between your nation and one troubled by insurgent violence. You notice a young man in a cheap car approaching the checkpoint with a determined look on his face. You suspect he means to cross the border in order to work illegally inside your country. He is rapidly approaching your station. Is it appropriate for you to shoot and kill the approaching man?”

**Car accident**

*Incongruent Dilemma Variant*

“You are driving through a busy city street when all of a sudden a young mother carrying a child trips and falls into the path of your vehicle. You are going too fast to break in time; your only hope is to swerve out of the way. Unfortunately, the only place you can swerve is currently occupied by a little old lady. If you swerve to avoid the young mother and baby, you will seriously injure or kill the old lady. Is it appropriate to swerve and hit the old lady in order to avoid the young mother and child?”

*Congruent Dilemma Variant*

“You are driving through a busy city street when all of a sudden a young mother carrying a child trips and falls into the path of your vehicle. You are going too fast to break in time; your only hope is to swerve out of the way. Unfortunately, the only place you can swerve is currently occupied by a group of children on their way to elementary school. If you swerve to avoid the young mother and baby, you will seriously injure or kill several of them. Is it appropriate to swerve and hit the schoolchildren in order to avoid the young mother and child?”

**Animal Research**

*Incongruent Dilemma Variant*

“You have been hired by a pharmaceutical company to conduct research on their products. Since products must be fit for human use, they are first tried out on animals. Your job is to find out the effects various chemicals have on rats, pigeons, rabbits, and monkeys. Most chemicals have only minor effects on the animals, but some cause them discomfort or even permanent damage. The chemicals you are researching are slated to form part of a new AIDS drug cocktail that will give new hope to millions of AIDS victims around the world. You anticipate saving many lives with the chemicals. Is it appropriate to test these chemicals on animals?”

*Congruent Dilemma Variant*

“You have been hired by a pharmaceutical company to conduct research on their products. Since products must be fit for human use, they are first tried out on animals. Your job is to find out the effects various chemicals have on rats, pigeons, rabbits, and monkeys. Most chemicals have only minor effects on the animals, but some cause them discomfort or even permanent damage. The chemicals you are researching are slated to form part of a new acne facial cleanser that will give new hope to people with pimples and greasy skin. You anticipate making many people feel better about their appearance with the chemicals. Is it appropriate to test these chemicals on animals?”

A2 Potential Mediators

*Cognitive Reflection Test*

The Cognitive Reflection Test (CRT) measures a respondent’s tendency to engage in Type-2 processing (Frederick 2005). The instrument consists of three questions which are easy to answer once the respondent actually thinks about them. However, each question suggests an intuitive answer, which is wrong. The CRT score simply counts the number of correct answers and varies between 0 and 3:

1. “A bat and a ball cost $1.10 in total. The bat costs $1.00 more than the ball. How much does the ball cost? _____ cents.“

(Correct response = 5 cents; Intuitive response = 10 cents)

1. “If it takes 5 machines 5 min to make 5 widgets, how long would it take 100 machines to make 100 widgets? _____ min.”

(Correct response = 5 min; Intuitive response = 100 min)

1. “In a lake, there is a patch of lily pads. Every day, the patch doubles in size. If it takes 48 days for the patch to cover the entire lake, how long would it take for the patch to cover half of the lake? _____ days.“

(Correct response = 47 days; Intuitive response = 24 days)

*Faith in Intuition*

Faith in Intuition (FI) describes respondent´s tendency to rely on Type-1 processes (Epstein et al. 1996). We use a German version of Epstein´s FI-measure from Keller et al. (2000), who add 5 items to the original scale. The instrument consists of 15 items, which the respondent rates on a 7-point scale ranging from “*completely false*” to *“completely true”*:

1. When I form an opinion about something, I rely entirely on my intuition. *
2. In most decisions it makes sense to rely on one's feelings. *
3. I am a very intuitive person.
4. I trust my initial feelings about people.
5. I trust my immediate reactions to others.
6. I believe in trusting my hunches.
7. The first idea is often the best. *
8. When it comes to trusting people, I can usually rely on my "gut feelings".
9. I can usually feel when a person is right or wrong even if I can't explain how I know.
10. My initial impressions of people are almost always right.
11. I am quick to form impressions about people.
12. When it comes to buying things, I often make decisions based on my gut. *
13. I can typically sense right away when a person is lying.
14. When I get lost, I usually decide spontaneously at road junctions in which direction to continue. *
15. I believe I can judge character pretty well from a person's appearance.

* = added by Keller et al. 2000

*Empathic Concern*

Empathic Concern (EC) assesses the extent to which the respondent experiences feelings of warmth, compassion and concern for the observed individual and is a subscale of Davis´ “Multidimensional Approach to Individual Differences in Empathy” (Davis 1980). We apply a German translation of the scale (Grimm 2015), which consists of 7 items, including 3 inverted items. The respondent rates the following items on a 8-point scale ranging from “*completely false*” to “*completely true*”:

1. I often have tender, concerned feelings for people less fortunate than me.
2. Sometimes I don't feel sorry for other people when they are having problems. (-)
3. When I see someone being taken advantage of, I feel kind of protective toward them.
4. Other people's misfortunes do not usually disturb me a great deal. (-)
5. When I see someone being treated unfairly, I sometimes don't feel very much pity for them. (-)
6. I am often quite touched by things that I see happen.
7. I would describe myself as a pretty soft-hearted person.

(-) = inverted items

*Moral Identity Scale*

In order to evaluate the self-importance of moral identity (MI), a concept introduced by Aquino et al. (2002), we used a German version from Merz and Tanner (2009). Aquino and Reed (2002: 1424) defined moral identity as „a self-conception organized around a set of moral traits“. The self-importance of moral identity scale (MI) consists of ten items, five items measure Internalization (I) and five items measure Symbolization (S). The first subscale, Internalization (e.g., “It would make me feel good to be a person who has these characteristics.”), captures the private dimension of moral identity and reflects the extent to which moral properties are central to the self-concept. The second subscale, Symbolization (e.g., “The fact that I have these characteristics is communicated to others by my membership in certain organizations.”), captures the public dimension of moral identity and describes the extent to which these traits are expressed through action. We assess moral identity (MI) as follows: First, the participants were asked to visualize a person with nine salient moral traits (e.g., *fair, friendly, helpful, hardworking, honest*). Then, participants indicated how well each of the following 10 statements described them on 5-point scales ranging from “*not true of me*” to “*completely true of me*”:

1. It would make me feel good to be a person who has these characteristics. (I)
2. I often wear clothes that identify me as having these characteristics. (I)
3. Being someone who has these characteristics is an important part of who I am. (S)
4. I would be ashamed to be a person who has these characteristics. (I), (-)
5. The types of things I do in my spare time (e.g., hobbies) clearly identify me as having these characteristics. (S)
6. The kinds of books and magazines that I read identify me as having these characteristics. (S)
7. Having these characteristics is not really important to me. (I), (-)
8. The fact that I have these characteristics is communicated to others by my membership in certain organizations. (S)
9. I am actively involved in activities that communicate to others that I have these characteristics. (S)
10. I strongly desire to have these characteristics. (I)

(I) Internalization

(S) Symbolization

(-) inverted items

A4 References

Aquino, K., and Reed, A. I. I. (2002). The self-importance of moral identity. J.Personal. Soc. Psychol. 83, 1423–1440. doi: 10.1037//0022-3514.83.6.1423

Conway, P., and Gawronski, B. (2013). Deontological and utilitarian inclinations in moral decision making: a process dissociation approach. J. Personal. Soc. Psychol. 104, 216–235. doi: 10.1037/a0031021

Davis, M. H. (1980). A multidimensional approach to individual differences in empathy. JSAS Catal. Select. Doc. Psychol. 10, 85–96.

Epstein, S., Pacini, R., Denes-Raj, V., and Heier, H. (1996). Individual differences in intuitive-experiential and analytical-rational thinking styles. J. Personal. Soc. Psychol. 71, 390–405. doi: 10.1037//0022-3514.71.2.390

Frederick, S. (2005). Cognitive reflection and decision making. J. Econ. Perspect. 19, 25–42. doi: 10.1257/089533005775196732

Grimm, J. (2015). Empathie-Messung nach Davis. Deutsche Version. Test-Dokumentation. MF-Working Paper 2015–03. Vienna: Methodenforum der Universität Wien

Keller, J., Bohner, G., and Erb, H.-P. (2000). Intuitive und heuristische Urteilsbildung–verschiedene Prozesse? Präsentation einer deutschen Fassung des „Rational-Experiential Inventory“ sowie neuer Selbstberichtskalen zur Heuristiknutzung. Zeitschrift für Sozialpsychologie 31, 87–101.

Merz, C., and Tanner, C. (2009). Weitere Befunde zur Validierung der Geschütze Werte Skala (GWS). KSPZ-Forschungsbericht Nr 2.
